# Patterns of Hybrid Loss of Imprinting Reveal Tissue- and Cluster-Specific Regulation

**DOI:** 10.1371/journal.pone.0003572

**Published:** 2008-10-29

**Authors:** Christopher D. Wiley, Harry H. Matundan, Amanda R. Duselis, Alison T. Isaacs, Paul B. Vrana

**Affiliations:** Department of Biological Chemistry, School of Medicine, University of California Irvine, Irvine, California, United States of America; Texas A&M University, United States of America

## Abstract

**Background:**

Crosses between natural populations of two species of deer mice, *Peromyscus maniculatus* (BW), and *P. polionotus* (PO), produce parent-of-origin effects on growth and development. BW females mated to PO males (bw×po) produce growth-retarded but otherwise healthy offspring. In contrast, PO females mated to BW males (PO×BW) produce overgrown and severely defective offspring. The hybrid phenotypes are pronounced in the placenta and include PO×BW conceptuses which lack embryonic structures. Evidence to date links variation in control of genomic imprinting with the hybrid defects, particularly in the PO×BW offspring. Establishment of genomic imprinting is typically mediated by gametic DNA methylation at sites known as gDMRs. However, imprinted gene clusters vary in their regulation by gDMR sequences.

**Methodology/Principal Findings:**

Here we further assess imprinted gene expression and DNA methylation at different cluster types in order to discern patterns. These data reveal PO×BW misexpression at the *Kcnq1ot1* and *Peg3* clusters, both of which lose ICR methylation in placental tissues. In contrast, some embryonic transcripts (*Peg10*, *Kcnq1ot1*) reactivated the silenced allele with little or no loss of DNA methylation. Hybrid brains also display different patterns of imprinting perturbations. Several cluster pairs thought to use analogous regulatory mechanisms are differentially affected in the hybrids.

**Conclusions/Significance:**

These data reinforce the hypothesis that placental and somatic gene regulation differs significantly, as does that between imprinted gene clusters and between species. That such epigenetic regulatory variation exists in recently diverged species suggests a role in reproductive isolation, and that this variation is likely to be adaptive.

## Introduction

Imprinted genes display allele-specific silencing based on parental origin. This phenomenon results in classes of genes with biases in expression of paternally-derived alleles as well as those preferentially transcribing maternally-derived alleles. These loci represent many gene families, and their products are involved in a variety of processes [1]. Misexpression of imprinted genes is associated with many diseases including numerous tumor types, growth dysplasias, neurological conditions, and several pregnancy-associated disorders [2]–[8]. Imprinted loci are found clustered in relatively discreet regions of mammalian genomes, implying common regulatory elements [9]. The allelic silencing of imprinted loci requires the establishment and subsequent erasure of germline-specific epigenetic marks (e.g. such that a paternally-derived allele may become maternally-derived in the following generation) [10].

The best-characterized of these gametic “imprints” are dense regions of methylated cytosine residues typically lying between imprinted loci and/or at promoters [11]. These regions are known as germline differentially methylated regions (gDMRs) [12]–[14], and survive the wave of demethylation that occurs during preimplantation mammalian development [15]–[17]. Germline DMRs are thought to be the primary imprint control regions (ICRs) for their associated domain. This is particularly true for those regions acquiring DNA methylation during spermatogenesis, as histones and their associated modifications are replaced by protamines [18]. Targeted deletions of gDMRs typically perturb imprinting status at the associated domain, usually dependent on which parent passes the targeted allele [19]–[21].

An increasing body of evidence indicates that these imprinting regulatory mechanisms are tissue-specific in multiple mammalian species [22]–[25]. These differences are particularly pronounced in comparisons of extra-embryonic vs. fetal tissues. Both individual imprinted genes and entire clusters display placenta-specific patterns of imprinting regulation [24]–[28]. Accordingly, imprinted loci have been shown to play major roles in placental development [26].

We have uncovered a naturally occurring animal model that mimics several aspects of imprinted gene associated disorders [27]. Hybrids between two recently diverged North American deer mice (genus *Peromyscus*) display asymmetric effects on growth and development. Female prairie deer (*P. maniculatus bairdii*; captive stock = BW) mated with male oldfield mice (*P. polionotus*; captive stock = PO) produce growth retarded offspring [28], [29]. Placentas produced by this cross (designated bw×po) are particularly affected, weighing ∼ half that of the parental strains [30]–[32].

Conversely, PO females mated to BW males produce dysmorphic overgrowth of placental and fetal tissues [30], [31], [33], [34], and display multiple defects reminiscent of imprinted gene disorders [27]. Between 10 and 15 percent of these PO×BW conceptuses lack visible embryonic structures, typically resembling placentas and associated membranes [27]. The majority of these PO×BW conceptuses are dead by late gestation; those that survive to parturition kill the mother due to inability to pass through the birth canal [27], [31].

Studies to date show perturbations of allelic usage and levels of imprinted gene expression are affected in the hybrids. The bw×po hybrids display only minor perturbations, largely confined to extra-embryonic tissues [27], [35], [36]. In contrast, the PO×BW offspring exhibit loss-of-imprinting (LOI) and/or significantly altered expression levels at most, but not all loci tested [27], [34]–[36]. The PO×BW LOI is mediated by a maternal effect [37]. To date, hybrid DNA methylation has only been examined at the *H19* locus, where loss was associated with bi-allelic expression. Here we 1. Further assess DNA methylation-imprinted gene expression correlations, 2.Examine imprinting at other clusters, 3. Assess imprinting patterns in placental, embryonic and CNS to discern potential patterns in the cluster/gene types affected.

## Results

The results are grouped by genomic region or tissue-type. We assessed at least four samples for each tissue/genotype combination in the allelic expression and at least two samples in each of the DNA methylation assays. Samples used in the latter assays are a subset of those used in the expression analyses. In the bisulfite sequence analyses, we did not include sequence reads with significant numbers of unconverted non-CpG cytosines (i.e. suggesting an incomplete reaction). See the methods section for locus-specific details.

### Hybrid misregulation of the *Kcnq1ot1* cluster

The *Kcnq1ot1* (formerly *Lit1*) domain contains a number of maternally-expressed genes in addition to the paternally-expressed *Kcnq1ot1* transcript. The single known gDMR in this region is maternally methylated across the *Kcnq1ot1* promoter and associated with its repression on that allele [38]. We have previously shown reduced expression of two linked maternally expressed genes *Cdknc1* and *Phlda2* (*aka Ipl*, *Tssc3*), in the PO×BW hybrids. To date, we have been unable to identify an expressed polymorphism in these genes to assess allelic expression.

**Figure 1:**
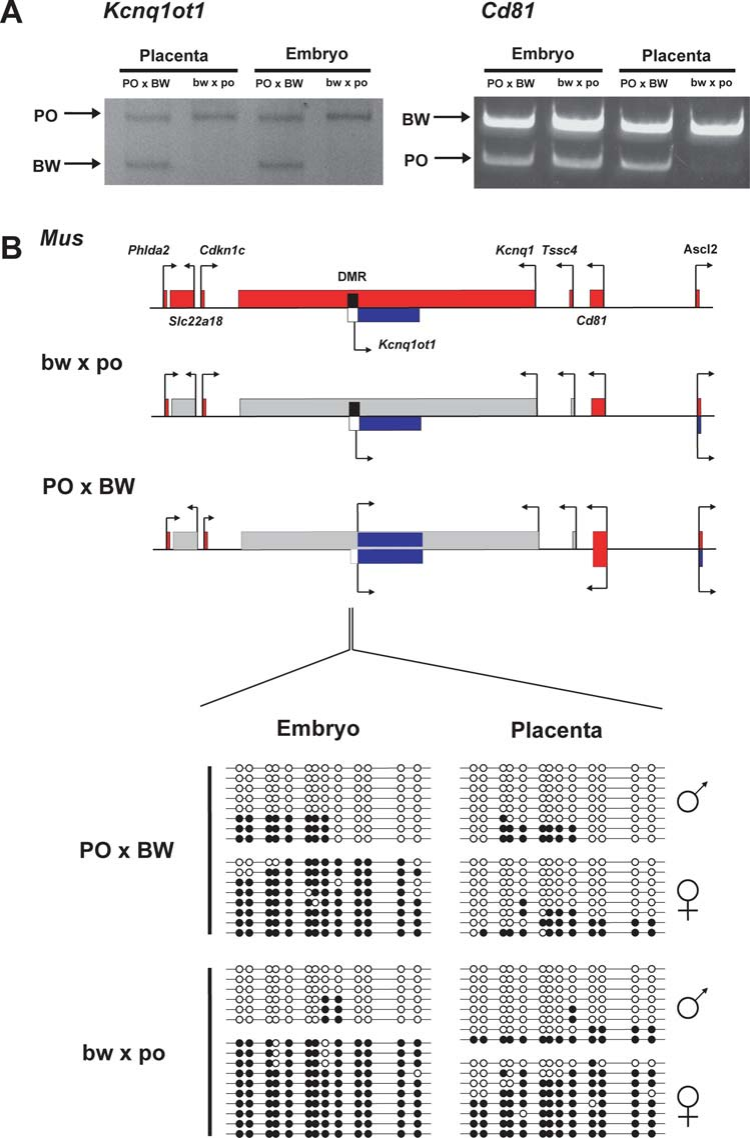
Disruptions in imprinting and DNA methylation at the Kcnq1ot1 domain. A. Allelic expression of the *Kcnq1ot1* and *Cd81* genes. An RT-PCR/RFLP assay is shown. Arrows indicate allele-specific bands. B. Domain structure and DNA methylation status as assessed by bisulfite sequencing. See text for details. *Mus* domain and imprinting status is shown at top. *Peromyscus* genes shown on same scale; complete genomic sequence was not available at the time of writing. Maternal allele expression indicated in red above line, paternal expression in blue below line. Grey–gene not examined in *Peromyscus*. gDMR is shown as a black (methylated) or white (unmethylated) box. Reactivated alleles are shown in their original color on the opposite allelic position. Sequenced clones from bisulfite-treated DNA shown at bottom. Each line represents an individual clone. Filled circles = methylated cytosines, open circles = unmethylated cytosines.

We were able to test imprinting status of another linked maternally expressed gene, *Cd81*(*Tapa1*), whose imprinting is limited to the placenta in house mice (*Mus*) [39]. The *Cd81* paternal allele is visible in bw×po placentas, but is clearly biased in favor of the maternal allele (Fig. 1A). Expression appears to be fully bi-allelic in the PO×BW placentas. While the *Cd81* data is consistent with the broad PO×BW LOI, this gene is not imprinted in cattle [40], raising the possibility that it may only be weakly imprinted in *Peromyscus*.

To determine if reactivation of the maternal *Kcnq1ot1* allele is associated with the misexpression in this cluster, we devised an allelic usage assay in the first (5′) kb of this long (∼50 kb) transcript [38]. Results of this assay indicate that *Kcnq1ot1* expression is biallelic in both PO×BW embryonic and placental tissues (Fig. 1A). In contrast, *Kcnq1ot1* displays strict paternal expression in bw×po tissues. These data are consistent with a model in which activation of the PO×BW maternal *Kcnq1ot1* transcript reduces *Cdkn1c* and *Phlda2* expression (as we have previously shown [27]). This suggests that the *Cd81* bi-allelic usage is more likely a lowering of maternal allele expression rather than activation of the paternal allele.

We performed bisulfite sequencing of the gDMR to determine whether the maternal *Kcnq1ot1* activation was associated with loss of DNA methylation on that allele. Parental origin of sequenced clones was determined by fixed PO-BW sequence polymorphisms. Both bw×po embryonic and placental tissues displayed clones that were either largely methylated or unmethylated (Fig. 1B). As expected, the methylated cytosines appeared largely on the silenced maternal BW allele. The correlation between methylation and expression was also evident in the PO×BW placentas. That is, the maternally derived PO clones typically displayed few methylated cytosines, reflecting the observed placental bi-allelic expression.

While the maternal *Kcnq1ot1* allele was also activated in PO×BW embryos, the bisulfite sequencing revealed little accompanying change in DNA methylation. While maintenance of *Kcnq1ot1* cluster imprinting has been shown to be regulated by epigenetic marks other than DNA methylation, this has been demonstrated primarily in extra-embryonic tissues [22]. The retention of *Kcnq1ot1* gDMR paternal methylation in PO×BW embryos may correlate with the earlier observation that *Phlda2* and *Cdkn1c* down-regulation is placenta-specific in the cross [27].

### Loss of methylation at the *Peg3-Usp29* domain

We have previously shown that the paternally-expressed *Peg3* gene displays LOI and increased expression in both embryonic and placental tissues. The neighboring *Usp29* gene also displays PO×BW LOI (data not shown). Imprinting of *Peg3* and *Usp29* is maintained in the bw×po hybrids [35]. We first undertook southern analysis with methylation-sensitive restriction enzymes to determine whether reactivation of the silenced allele is accompanied by alterations in DNA methylation at the gDMR location identified in *Mus*. Through a length polymorphism, these data did indeed indicate loss of methylation from the maternal allele (Figure S1).

**Figure 2:**
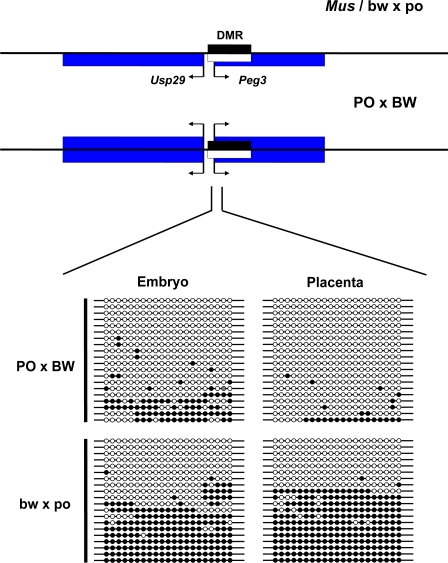
Loss of methylation at the *Peg3-Usp29* locus. Top–Locus structure/expression in *Mus* and reciprocal hybrids. All symbols are as described in Figure 1. Bottom–Bisulfite sequencing of the ICR in *Peromyscus* crosses. No polymorphism was available to determine parental origin in the bisulfite reads.

We next performed bisulfite mutagenesis and sequencing of the *Peg3* gDMR to more thoroughly examine individual gDMR cytosine methylation status. While there is no PO-BW polymorphism in the amplified region, both embryonic and placental bw×po clones displayed the expected 50∶50 methylated (maternal): unmethylated (paternal) ratio (Fig. 2). In contrast, both PO×BW tissues displayed a large proportion of unmethylated sequence reads, with a greater proportion in the placenta. Thus these data indicate a correlation of DNA methylation loss at the ICR with reactivation of the *Peg3* maternal allele.

### Tissue-specific loss of imprinting with little DNA methylation loss at *Peg10*

**Figure 3:**
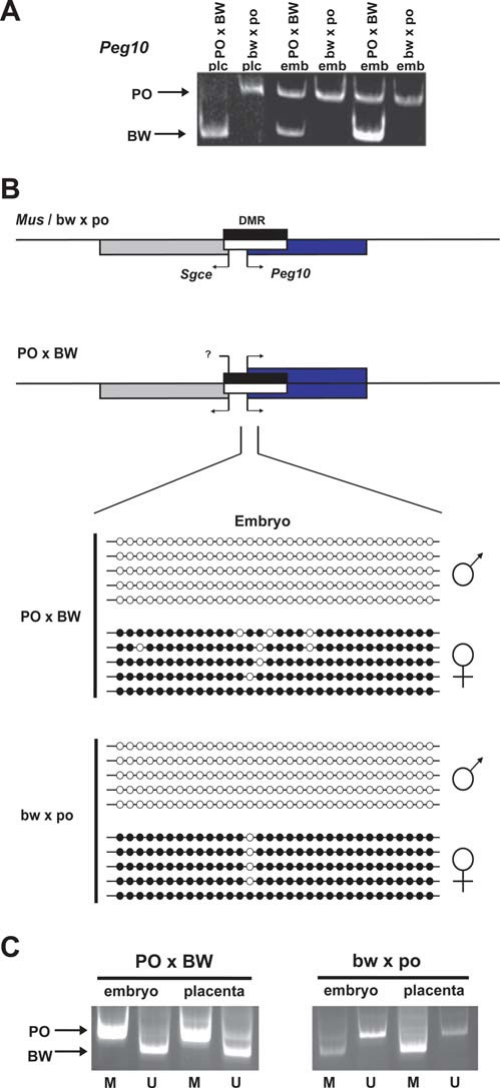
Imprinting and methylation analysis of the *Peg10-Sgce* domain. A. Allelic expression of the Peg10 transcript. Arrows indicate allele-specific bands. B. Domain structure and DNA methylation status as assessed by bisulfite sequencing. The region assayed starts in the intergenic region and extends several hundred base-pairs into *Peg10* intron 1. All symbols are as described in Fig. 1. C. Methyl-specific PCR (MSP) assay. Primers were designed to amplify either methylated or unmethylated bisulfite-treated DNA. Methylated and unmethylated products are indicated with M and U (respectively) below each lane. The amplicons are from sequences ∼200 bp 5′ of the bisulfite assay results shown (∼300 bp 5′ of the *Peg10* transcription start site).

The *Peg10-Sgce* cluster is organized a similar manner to the *Peg3-Usp29* pair, with a gDMR lying between the two oppositely oriented paternally expressed loci. To test *Peg10* allelic expression, we devised an RT-PCR/RFLP assay. Results of this assay indicated that *Peg10* LOI is limited to PO×BW embryonic tissues. This is the first time this combination (placental imprinting; embryonic LOI) has been observed in the offspring of this cross (Fig. 3A). The bw×po hybrids again displayed imprinted *Peg10* expression in both somatic and extra-embryonic components. Despite sequencing over 2 kb of the *Sgce* gene, no PO-BW polymorphism was detected to assess allelic expression.

We next performed bisulfite sequence analysis to determine if loss of cytosine methylation at the intergenic gDMR correlated with the *Peg10* LOI observed in PO×BW embryos (Fig. 3B). Due to difficulty cloning the ∼1 kb *Peg10* gDMR amplicons, we supplemented this analysis with a methylation-specific PCR (MSP) assay. In this assay, separate primer sets are designed for the converted vs. unconverted strands after bisulfite treatment. A length polymorphism in the amplified region allowed determination of allelic parental origin (Fig. 3C).

As expected, both assays revealed high levels of methylation on the *Peg10* maternal allele and little methylation on the paternal allele in all cross/tissue combinations. In both cases, there were possible indications of minor methylation loss from the PO×BW maternal allele.

### Lack of perturbations at the *Meg3-Dlk1* domain

Several studies have suggested structural similarities between this domain and the *H19*-*Igf2* pair. Both domains contain a maternally-expressed (*H19*, *Meg3*) and a paternally expressed (*Igf2*, *Dlk1*) locus separated by a paternally methylated IG-DMR/ICR [41]. The biallelic expression of *H19* (but imprinting of *Igf2*) in PO×BW offspring [35] might then imply similar deregulation of *Meg3* (formerly *Gtl2*) but imprinting of *Dlk1*. Consistent with this hypothesis, *Dlk1* was shown to be paternally expressed in the PO×BW offspring as the result of a screen for novel imprinted loci [42].

**Figure 4:**
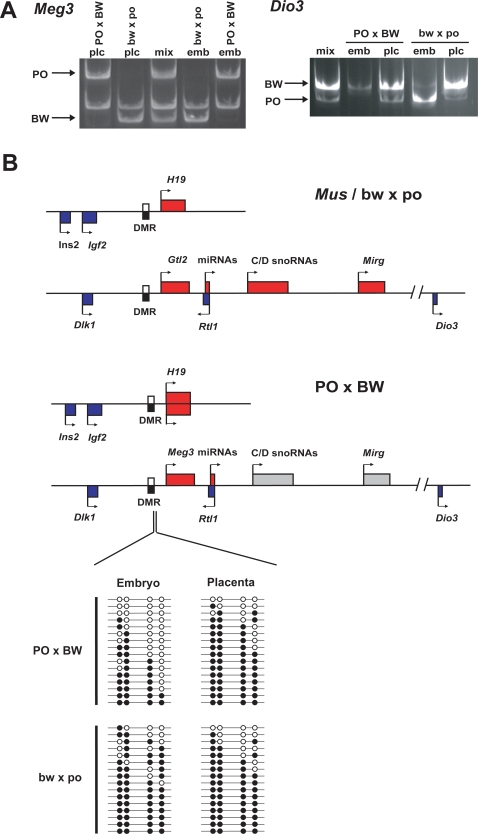
Imprinting analysis of the *Dlk1-Meg3* imprinted domain. A. Allelic usage assays for *Meg3* and *Dio3*. F1 DNA is included to demonstrate an allelic amplification bias in biallelic samples of *Dio3*. *Gtl2* is ubiquitously imprinted in both hybrids, while *Dio3* displays embryo-specific imprinting. B. Structure of *Igf2-H19* and *Dlk1- Meg3* domains and differences in imprinting perturbations in PO×BW hybrids. C. Bisulfite sequencing of the domain gDMR. Symbols as in Fig. 1.

To determine if the *Meg3* locus loses imprinting analogous to that observed at *H19*, we developed an RT-PCR/ RFLP assay to determine allelic usage. Unlike *H19*, *Meg3* remains tightly imprinted in the placentas and embryos of both crosses (Fig. 4A). Another transcript in this domain, *Dio3*, displays preferential expression of the paternal allele in *Mus* embryos [43], but is not imprinted in placental tissues. We examined *Peromyscus* hybrid *Dio3* allelic expression by a similar assay. Similar to *Mus*, this gene displayed biallelic expression in both hybrid placenta types, but was imprinted in embryonic tissues (Fig. 4A). These data demonstrate that, unlike the *Igf2*-*H19* domain, imprinting in the *Dlk1*-*Meg3* imprinted domain is not significantly influenced by interspecific hybridization.

We investigated whether DNA methylation might be perturbed at this locus in spite of the apparently undisturbed imprinting status. We carried out bisulfite sequence analysis on a portion of the 3′ end of the *Meg3* gDMR. The methylation patterns were not clearly allelic, and no polymorphism was available to determine parental origin. There was no unambiguous evidence for loss of methylation in this region (Fig. 4B). However, three of sixteen PO×BW embryonic clones displayed no methylated cytosine residues while this was not true of any of the bw×po derived clones.

### Allelic expression of the *Plagl1* and *Dcn* imprinted loci

To further characterize the hybrid imprinting patterns, we assessed the allelic expression status of two imprinted genes not linked to any other of those investigated. The *Plagl1* gene (aka *Zac1*, *Lot1*) has an associated gDMR and one additional linked imprinted locus (HYMAI), but its regulation has been little studied [44], [45]. The Decorin (*Dcn*) gene lacks any other known associated imprinted loci or gDMR [46]. The *Mus Plagl1* gene is paternally expressed in several fetal and adult tissues, as is its human ortholog in placentas [47], [48]. *Dcn* has been shown to be maternally expressed in *Mus* placentas, but exhibits biallelic expression in other tissues [49] and other species (cows, humans) [50], [51].

**Figure 5:**
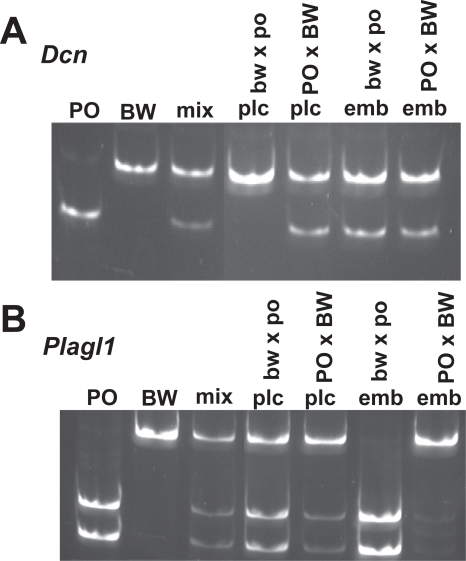
Allelic expression of the *Dcn* (A) and *Plagl1* (B) genes. The patterns for the PO, BW and a mix of the two alleles are shown at left. Lane identity at top; plc–placenta, emb–embryo. Note that other PO×BW samples displayed reactivation of the *Plagl1* maternal allele.

Similarly, analysis of bw×po hybrid placentas shows imprinted expression of *Dcn*, and biallelic expression in all other tissues (Fig. 5A). *Dcn* is biallelic in all PO×BW tissues, suggesting that imprinting is lost in the placenta. Placental expression of *Plagl1* is also consistently biallelic in the in PO×BW hybrids. As this is also true in the case of the bw×po hybrids, this likely also represents differences in embryonic vs. placental regulation. Note, however, that a slight amplification bias in favor of the BW allele indicates that the bw×po placentas may be closer to imprinted than those from the PO×BW cross. Allelic expression is more sporadic in PO×BW embryonic tissues, with some samples exhibiting strict imprinting (Fig. 5B), and others bi-allelic expression. Thus, the *Dcn* and *Plagl1* data further suggest tissue-specific differences in hybrid misregulation of imprinted loci.

### Brain-specific imprinting effects

Previous studies in other mammalian groups suggest that the brain represents another instance of tissue-specific regulation of imprinted loci. For example, the *Igf2* gene exhibits biallelic expression in the brains of house mice, humans, and *Peromyscus* [30], [39]–[41]. To assess whether the hybrid misregulation also exhibited brain-specific patterns, we tested the allelic expression of several imprinted genes including *H19*, *Peg10*, *Kcnq1ot1*, *Plagl1*, and *Meg3*.

**Figure 6:**
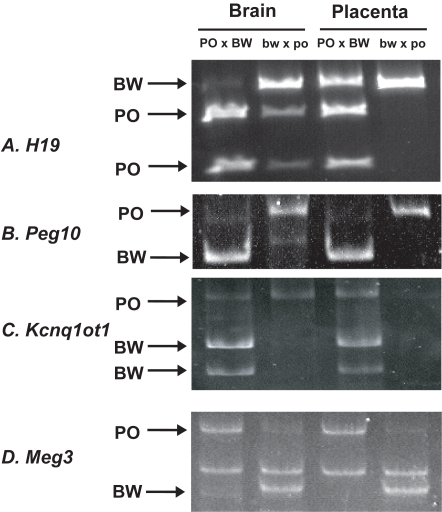
Allelic expression in the brains of *Peromyscus* hybrids. Placental expression is shown for comparison. Arrows indicate allele-specific bands. A–*H19*; B–*Peg10*; C–*Kcnq1ot1*; D–*Meg3*.

Unlike placental and other embryonic tissues, the brains of PO×BW embryos displayed maternal expression of *H19* (Fig. 6A). Equally surprisingly, bw×po brains exhibited some reactivation of the paternal *H19* allele, despite strict imprinted expression elsewhere. The *Peg10* gene also showed paternal expression in the PO×BW hybrids examined (Fig. 6B), in contrast to that observed in whole embryos. The bw×po brains also displayed imprinted *Peg10* expression.

In contrast, *Kcnq1ot1* appears to be universally imprinted in bw×po conceptuses and to lose imprinting in all tissues of PO×BW conceptuses (Fig. 6C). Similarly, the *Meg3*, *Plagl1* and *Peg3* brain allelic expression patterns also resemble the expression patterns of the fetus as a whole (Fig. 6D and not shown).

## Discussion

A logical approach to understanding the mechanisms of genomic imprinting is to categorize gene clusters by apparent regulatory similarities. Examples include allele-specific boundaries (*H19*/*Igf2*), functional ncRNAs (e.g. *Kcnq1ot1*), and dual promoter methylation (e.g. *Peg3*/*Usp29*). The patterns of imprinting perturbations in the *Peromyscus* hybrids (summarized in Table 1) suggest functional differences in several of these analogous clusters. For example, evidence suggests that the maternally expressed *Igf2*r and *Cd81* loci are each regulated by linked paternally-expressed ncRNAs (*Air* and *Kcnq1ot1*, respectively) [52], [53]. *Igf2*r retains normal imprinted maternal expression in PO×BW hybrid crosses [35], while the *Kcnq1ot1-Cd81* cluster is misregulated. In contrast, the paternal allele of *Igf2r* is reactivated in bw×po placentas [35] while both *Cd81* and *Kcnq1ot1* are unperturbed in this cross.

**Table 1:** Summary of hybrid imprinted gene expression.

Gene	Imprint Mechanism	PO×BW			bw×po			Ref
		Plac	Emb	CNS	Plac	Emb	CNS	
*Igf2*	ICR/Allele-specific insulator	Pat	Pat	Bi	Pat	Pat	Bi	†, [35], [27]
*H19*	ICR/Allele-specific insulator	Bi	Bi	Mat	Mat	Mat	Bi	†, [35], [33]
*Igf2r*	ICR/ncRNA	Mat	Mat	Bi	Bi	Mat	Bi	[35]
*Mest*	gDMR	Bi	Bi	ND	Pat	Pat	ND	[35]
*Snrpn*	ICR/ncRNA (?)	Bi	Bi	ND	Pat	Pat	ND	[35]
*Peg3*	gDMR	Bi	Bi	Bi	Pat	Pat	Pat	[35], [78], [33]
*Usp29*	gDMR	Bi	Bi	ND	Pat	Pat	ND	†
*Peg10*	gDMR	Pat	Bi	Pat	Pat	Pat	Pat	†
*Dlk1*	gDMR	Pat	Pat	ND	Pat	Pat	ND	[42]
*Meg3*	gDMR	Mat	Mat	Mat	Mat	Mat	Mat	†
*Dio3*	gDMR	Bi	Pat	ND	Bi	Pat	ND	†
*Plagl1*	gDMR	Bi	Pat	Pat	Bi	Pat	Pat	†
*Phlda2*	ICR/ncRNA	Δ↓	∼par	ND	∼par	∼par	ND	[27]
*Cdkn1c*	ICR/ncRNA	Δ↓	∼par	ND	∼par	∼par	ND	[27]
*Kcnq1ot1*	ICR/ncRNA	Bi	Bi	Bi	Pat	Pat	Pat	†
*CD81*	ncRNA	Bi	Bi	ND	Mat	Bi	ND	†
Ascl2*	ncRNA	Bi	Bi	NA	Bi	Bi	NA	[35]
*Grb10*	gDMR/ND	Bi	Bi	ND	Bi	Mat	ND	[35], [27]
*Gatm*	(?)	Mat	Mat	ND	Mat	Mat	ND	[79]
*Dcn*	(?)	Bi	Bi	Bi	Mat	Bi	Bi	†
Rasgrf1*	ICR/Allele-specific insulator (?)	ND	ND	ND	Bi	Bi	ND	[80]
*Xist*	ncRNA	Pat	BW	ND	Pat	BW	ND	[32]

Plac–placenta; emb–embryo; CNS–brain; Ref–study. Pat–paternal expression; Mat–maternal expression; Bi–biallelic expression; Δ↓–reduced expression; ∼par–expression equivalent to parental strains. †-this study; * *Ascl2 & Rasgrf1* do not appear to be imprinted in *Peromyscus*. Mechanisms are adapted from [81]. ND–not determined. NA–not applicable due to lack of expression.

Similarly, the loss of *H19* imprinting in PO×BW crosses is not mirrored by its analogue *Meg3*, which retains maternal mono-allelic expression. Differences in hybrid regulation are also seen in the *Peg10* and *Peg3* domains, despite the apparent similarities in structure and regulation. While both domains exhibit loss-of-imprinting in the PO×BW cross, DNA methylation at *Peg10* is apparently little perturbed.

These data suggest similarities in *Peg3*, *Kcnq1ot1* and *H19* imprinting cluster regulation not shared by more putatively similar loci (e.g. *Peg 10*, *Meg3*). We hypothesize that a major commonality among these loci will be regulation by the product of the maternal effect locus (*Meil*) for which genetic evidence has previously been described [37]. Specifically, we suggest that the PO allele of this gene cannot initiate and/or sustain imprinting in the presence of BW chromatin (i.e. PO×BW cross). Demonstrated imprinting within *P. polionotus* implies that there is natural variation at this locus, rather than a null allele or absence of the locus in this species [35]. Identification of this locus is therefore paramount in understanding the hybrid misregulation as well as normal epigenetic regulation of mammalian development.

We have shown that *Meil* is not identical to any of the DNA methyltransferase (*Dnmt*) loci, nor to several other candidate loci [33]. We suggest that the *Meil* product is deposited in oocytes and involved in epigenetic regulation. This hypothesis is strengthened by a study of reciprocal *in vitro* fertilization efficiency between PO and BW [54]. This study found that 8% of BW oocytes fertilized with PO sperm resulted in abnormalities (i.e. failure to fertilize or cleave). In contrast, PO oocytes fertilized with BW sperm resulted in a 26% abnormality rate [54].

There are two potential caveats to the observed patterns of imprinted gene misregulation. First, while we have documented imprinting within *P. polionotus*, it is formally possible that some loci are not imprinted in *Peromyscus* but bias is induced in the bw×po or both hybrid types. If this is the case, it may be a widespread problem. The house mouse crosses used to define many imprinted loci involve crosses to other species or subspecies that have also been associated with decreased fitness [55]–[57]. More broadly, crosses between species/strains that exhibit many expressed polymorphisms (used for ease of developing allelic expression assays) may also be polymorphic at *cis*-regulatory sequences.

The second potential caveat lies in our examination of tissues from the second half of gestation; prior analyses suggest that the majority of PO×BW conceptuses are dead by this time [27]. Other studies suggest a stochastic element in the loss of epigenetic marks [58], which is also suggested by the patterns of DNA methylation loss in PO×BW conceptuses (e.g. *Peg3*; Fig. 2). This suggests the possibility that perturbations are typically greater in this cross, but that we have selected for less affected conceptuses. For example, *Peg10* has been shown to be necessary for *Mus* placental development [59]. Strict imprinting of *Peg10* in the examined PO×BW conceptuses may reflect that over-expression is similarly incompatible with survival to late gestation. A somatic example of *Peg10* loss-of-imprinting has been found in several tumor types [60], [61].

One possibility is that the PO×BW imprinting perturbations in particular are byproducts of more inclusive epigenetic perturbations. For example, DNA methylation is now known to regulate many non-imprinted/X-linked loci [62], [63]. Consistent with the variability seen in the PO×BW cross, deletion of even the oocyte-specific version of the maintenance DNA methyltransferase Dnmt1 (DNMT1o) also results in a large variety of defects [58]. The knockout variation is thought to be due to stochastic epigenetic variation present in the early embryo.

A stochastic element to PO×BW epigenetic mark loss may also be an important element in understanding the hybrid defects. We hypothesize that cells which lose sufficient DNA methylation at the *Kcnq1ot1* and similar ICRs result in androgenetic-like expression patterns. Androgenetic (no maternally inherited genome) *Mus* embryos and typical androgenetic hydatidiform moles result in an early embryonic shift towards extra-embryonic cell fates. In this scenario, the PO×BW loss of DNA methylation is not due to placenta-specific factors; rather, loss of methylation results in a gene expression profile more compatible with extra-embryonic fates.

Several aspects of the PO×BW cross recall those observed in human biparental hydatidiform moles (BiHMs). Hydatidiform moles are typically androgenetic (e.g. due to loss of the oocyte pronucleus) [64]. In the last decade, molar pregnancies with a normal 1∶1 parental genomic contribution have been reported [65], [66], and are thought to account for the majority of recurrent familial cases of this syndrome [67]. In at least several of these families, women have borne normal children between BiHM pregnancies [67], [68]. This suggests negative interactions between variants in an oocyte-associated gene product and elements within the sperm.

BiHM tissues exhibit complex patterns of imprinted gene expression and methylation perturbations via a maternal effect locus or loci [66], [68], [69]. These loci include *PEG3*, *SNRPN*, *KCNQ1OT1*, *CDKN1C* [69], [70], and in some cases *H19* [70]. Genetic studies indicate the existence of multiple BiHM susceptibility loci [67]. One of these loci maps to human chromosome 19q13.4 [71], the equivalent domain to which a paternal expressed gene involved in the *Peromyscus* placental hypertrophy maps [72]. While *Peg3*/*PEG3* lies in this region, no human coding SNPs of the gene correlate with BiHM susceptibility. Instead, the *NLRP7* (formerly *NALP7*) gene has emerged as the primary candidate in this region [69], [73].

The NLRP7 product is known to be involved in apoptotic and inflammatory pathways, but the connection to the molar phenotype is unclear. House mice apparently lack a NLRP family member in this region; the *Peromyscus* domain has not yet been characterized. It is striking that the two phenomena (BiHM & PO×BW hybrids) display imprinting perturbations, trophoblast over-proliferation, and a genetic component mapping to corresponding genomic regions. Both identification of *Meil* and characterization of the early PO×BW epigenetic misregulation are likely to aid in understanding these profound shifts in cell-fate.

That such epigenetic regulatory variation exists in recently diverged species suggests a role in reproductive isolation, and that this variation is likely to be adaptive. Epigenetic phenomena by definition mediate gene-environment interactions. The importance of such interactions is increasingly being recognized in understanding biological processes. We believe this system, having the potential to link naturally differing allelic combinations with environmental and behavioral variation, has a unique potential to aid in elucidating these interactions [74].

## Methods

### Animals and Breeding

We purchased PO and BW stocks from the *Peromyscus* Genetic Stock Center (http://stkctr.biol.sc.edu/). Both parental strain and interspecific cross conceptuses were bred at UCI. Animals were kept and treated in conditions approved by the University of California Irvine Institutional Animal Care and Use Committee (IACUC), protocol #2001-230. Animals were fed a standard *ad libitum* high protein/fiber diet and water. The light/dark cycle was 16∶8 hours. Hybrid embryos were collected at the equivalent of *Mus* embryonic14.5 for all assays. Embryos and placentas were split sagittally, with one half harvested for RNA, and the other for DNA.

### Cloning of *Peromyscus* Sequences

Human, mouse, and/or other available mammalian species were aligned, and primers for the cloning of imprinted genes/DMRs from *Peromyscus*. Primers were designed to conserved regions when possible. To acquire *Peromyscus* ICR/gDMR sequences, we probed a *P. maniculatus* BAC library filters (CHORI-Oakland) with PO/BW imprinted gene sequences. Positive clones were then ordered from CHORI and confirmed via PCR. Relevant DMR sequences were then cloned via PCR using BAC clones as templates, followed by primer walking. When this was not possible, BACs were sequenced to acquire sequence from the relevant regions. All sequences have been deposited in Genbank, accessions numbers EU746661-EU746681.

### Allelic Expression Assays

Prenatal tissues were harvested at the equivalent of *Mus* embryonic day 14.5. Heads of embryos were removed prior to RNA extraction in order to avoid brain-specific assay complications. RNA was isolated from embryos, placentas, and brains with the Qiagen RNeasy kit, including a DNase step to remove genomic DNA. Superscript II reverse transcriptase (Invitrogen) was used to generate cDNA. For *Kcnq1ot1*, a series of *Kcnq1ot1*-specific oligonucleotides were used as primers for cDNA synthesis. In all other cases, oligo-dT was used for synthesis. RT(-) reactions were used as controls against gDNA contamination. Allelic usage was determined by restriction fragment length polymorphism. Mixes of parental strain templates were used to assess possible allelic amplification bias [75]. Primers and annealing temperatures are listed in Table S1.

### DNA Methylation Analysis via Bisulfite Treatment and Sequencing

DNA was isolated from *Mus*-equivalent e14.5 embryos and placentas via either phenol extraction or Qiagen DNeasy kit. Isolated DNA was then subject to bisulfite conversion via agarose bead [76] or Methylamp™ DNA Modification Kit (Epigentek) protocols. Approximately 400 ng of bisulfite-converted DNA was used per PCR reaction. PCR mixes contained 1.5 mm MgCl2, 1× PCR buffer (Applied Biosciences), 1 U Taq polymerase, and 100 pg each primer. Primers were designed from cloned sequences via Methprimer [77], and were used in nested reactions. Primary PCR reactions featured 35–40 cycles, followed by a 20 cycle nested reaction. Primers and Annealing temperatures are listed in Table S1.

Successful PCR products were cloned into a TOPO-TA vector (Invitrogen) and sequenced. As noted, sequence reads that suggested incomplete bisulfite reactions were discarded. In the case of the *Peg3* and *Kcnq1ot1* DMRs no sequence read with more than 3 unconverted non-CpG cytosines was included in the analysis. For the *Meg3* DMR, no read with more than 2 was included. For the *Peg10* DMR, no read with more than 10 was included (the region analyzed contains 321 cytosine residues).

### Methylation Specific PCR (MSP)

To determine allelic methylation within the *Peg10* gDMR, we designed primers specific for methylated and unmethylated bisulfite-treated DNA. The assay primers span a region where a PO/BW size polymorphism allows determination of parental origin for amplification products. MSP reactions were performed using the first round of *Peg10* bisulfite PCR products as templates, and were carried out over 20 cycles. Primers and annealing temperatures are listed in Table S1.

### DNA methylation Analysis via Restriction Digestion/Southern Analysis

DNA was extracted via phenol extraction and ethanol precipitation. Ten to fifteen micrograms of each extract were digested with *Hpa*II, *Msp*I and or *Eco*RI as indicated (New England Biolabs). Digests were then electrophoresed on 1% agarose gels. For *Peg3* southern analysis, the probe was random hexamer oligo-labelled and hybridized in Church buffer at 65°C. Washes were done at 65°C in 0.5× SSC, 0.1% SDS.

## Supporting Information

Figure S1 Southern blot analysis of PO×BW DNA methylation at the Peg3 locus. Genomic DNA was first digested with EcoRI (RI) alone, then divided into 3 aliquots. The first was not treated further; the other 2 were subsequently digested with MspI (+Msp) or HpaII (+Hpa). The probe used was a ∼580 bp fragment corresponding to sequence from the Peg3/Usp29 intergenic region to Peg3 intron1. Genotype is listed at top; PO DNA is shown with all 3 enzyme combinations. BW DNA is shown cut with RI alone to illustrate the species size polymorphism (PO∼11 kb, BW∼16 kb). Arrows at side indicate either allele-specific bands or fully digested RI+Msp DNA (Dig). Note in the PO×BW+Hpa lane that the paternal BW allele is absent, and that the maternal (PO) band is also severely reduced.
(2.05 MB TIF)

Table S1 Details of PCR-based assays. Alleles-allele usage (imprinting) assays; Bisulfite-bisulfite treated DNA sequence assays; MSP-methylation-specific PCR assays. Gene name indicated in this column. 1°-primary PCR; 2°-secondary (nested) PCR. M-methylated alleles; U-unmethylated alleles. Tmp-annealing temperature for indicated PCR; Enzyme-restriction endonuclease used to cleave amplicons; BW, PO frag(s)-fragments generated by assay for those genotypes (in base pairs).
(0.06 MB DOC)
