# Supplementary figures and images for: Patterns of Hybrid Loss of Imprinting Reveal Tissue- and Cluster-Specific Regulation
Source: PLoS One. 2008 Oct 29;3(10):e3572. doi: 10.1371/journal.pone.0003572 (PMC2570336; doi:10.1371/journal.pone.0003572)

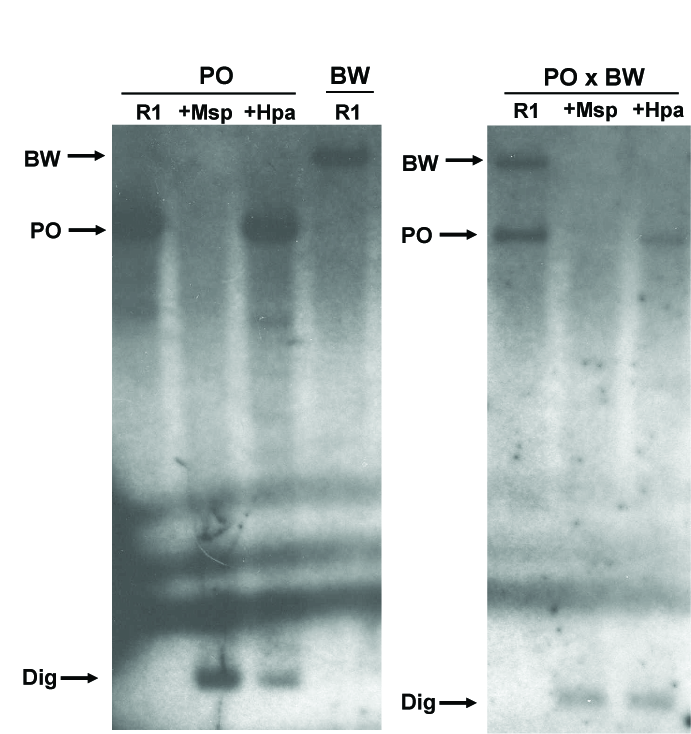

Supplement: Figure S1 — Southern blot analysis of PO×BW DNA methylation at the Peg3 locus. Genomic DNA was first digested with EcoRI (RI) alone, then divided into 3 aliquots. The first was not treated further; the other 2 were subsequently digested with MspI (+Msp) or HpaII (+Hpa). The probe used was a ∼580 bp fragment corresponding to sequence from the Peg3/Usp29 intergenic region to Peg3 intron1. Genotype is listed at top; PO DNA is shown with all 3 enzyme combinations. BW DNA is shown cut with RI alone to illustrate the species size polymorphism (PO∼11 kb, BW∼16 kb). Arrows at side indicate either allele-specific bands or fully digested RI+Msp DNA (Dig). Note in the PO×BW+Hpa lane that the paternal BW allele is absent, and that the maternal (PO) band is also severely reduced. (2.05 MB TIF) [file pone.0003572.s001.tif]
