# Supplementary material for: Patterns of Hybrid Loss of Imprinting Reveal Tissue- and Cluster-Specific Regulation
Source: PLoS One. 2008 Oct 29;3(10):e3572. doi: 10.1371/journal.pone.0003572 (PMC2570336; doi:10.1371/journal.pone.0003572)
Supplement: Table S1 — Details of PCR-based assays. Alleles-allele usage (imprinting) assays; Bisulfite-bisulfite treated DNA sequence assays; MSP-methylation-specific PCR assays. Gene name indicated in this column. 1°-primary PCR; 2°-secondary (nested) PCR. M-methylated alleles; U-unmethylated alleles. Tmp-annealing temperature for indicated PCR; Enzyme-restriction endonuclease used to cleave amplicons; BW, PO frag(s)-fragments generated by assay for those genotypes (in base pairs). (0.06 MB DOC) [file pone.0003572.s002.doc]

| **Alleles** | **Forward Primer** | **Reverse Primer** | **Tmp** | **Enzyme** | **BW Frag(s)** | **PO Frag(s)** |
| --- | --- | --- | --- | --- | --- | --- |
| *Gtl2* | TGGATTATGCGGCAAATGGA | AGTCAGGAAGCAGTGGGTTG | 59ºC | NlaIII | 221, 173, 163 | 336, 221 |
| *Peg10* | TGGCATCTACACACGGACAT | TAGAGCATAGTACACACTGCCAAA | 59ºC | MseI | 144, 32 | 176 |
| *Cd81* | TGCTCTTCGTCTTCAATTTCG | GTTGAAGGGCCTGGTCATAG | 58ºC | Fnu4HI | 280, 80 | 215, 80, 65 |
| *Lit1* | ATGTTGGAGGGAGGGGTATC | CCTTTCACAGGGGTCACCTA | 59ºC | HpyCH4III | 197, 132 | 329 |
| *Zac1* | GTTCAACCGCAAGGACCAC | TCTTGGTGTGACGAGTGAGG | 57ºC | DdeI | 407 | 232, 175 |
| *Dcn* | GTCGTCTACCTTCATAACAA | GGCAGAGCGCACGTAGA | 49ºC | MboI | 128, 42 | 90, 42, 38 |
| *Dio3* | ATCCGCAAGCATTTCCTG | GTAGGCGAATCTGAGGTGA | 59ºC | DdeI | 444 | 399, 45 |
| *Ocat* | TAGCTTAGTGCTTTGGTGTTGC | AGGGAGTTGACGTCAACGGAGT | 55ºC | HphI | 320 | 200, 120 |
|  |  |  |  |  |  |  |
| **Bisulfite** |  |  |  |  |  |  |
| *Lit1* 1° | ATTTTGATTATAGAGGTAGGGGGTG | TAATACCCCTCCCTCCAACATATA | 59ºC | N/A | 273 | 273 |
| *Lit1* 2° | GGGGTGGTTTTGAGGTTAGTT | CCAACATATAAAAACATCCCAAAC | 59ºC | N/A | 249 | 249 |
| *Peg3* 1° | TTGTTTTGGTAATTGTAGTTTGATTG | CAAAAAATACCCCTCAAATCTTAAC | 59ºC | N/A | 550 | 550 |
| *Peg3* 2° | TTGTAGAGGATTTTGATAAGGAGGT | ATTTTTACAACCTTATCAATCACCC | 59ºC | N/A | 360 | 360 |
| *Peg10* 1° | TTTTTTTTAGTTTGGTTAGTTTAGTAT | TCCAAAACCACTTTTTCTAAAACTC | 56ºC | N/A | 1228 | 1251 |
| *Peg10* 2° | GGGTTGTATTTTTTATTTATTTTTT | CTTTTACATTTAATTACTCTCCTACACTCA | 56ºC | N/A | 1045 | 1075 |
| *Gtl2* 1° | TTTGTGTTTTGAGATAATAGGTTATTTATT | AAATTTCCTAAACTTAAAATCAACCC | 54ºC | N/A | 225 | 225 |
| *Gtl2* 2° | TTGAGATAATAGGTTATTTATTATTTTT | AATCAACCCATTCACTAAAAACC | 52ºC | N/A | 201 | 201 |
|  |  |  |  |  |  |  |
| **MSP** |  |  |  |  |  |  |
| *Peg10* M | TATATGAGTTACGGATCGAATTGC | TAATACAATAAAACGCATTAAACGC | 59ºC | N/A | 264 | 294 |
| *Peg10* U | ATATGAGTTATGGATTGAATTGTGA | AATACAATAAAACACATTAAACACC | 53ºC | N/A | 264 | 294 |
